# Serum adiponectin level is negatively related to insulin resistance in women with polycystic ovary syndrome

**DOI:** 10.1530/EC-24-0401

**Published:** 2024-12-13

**Authors:** Jie Yang, Min Lin, Xiaoyan Tian, Chujun Li, Haocun Wu, Ling Deng, Xuelan Li, Xin Chen

**Affiliations:** ^1^ Reproductive Medicine Center, Shunde Hospital of Southern Medical University (The First People's Hospital of Shunde), Foshan, Guangdong, China; ^2^ Reproductive Medicine Center, The First People’s Hospital of Yulin, Yulin, Guangxi Zhuang Autonomous Region, China; ^3^ Department of Clinical Laboratory, Shunde Hospital of Southern Medical University (The First People's Hospital of Shunde), Foshan, Guangdong, China

**Keywords:** polycystic ovary syndrome, insulin resistance, adipokines, adiponectin to leptin ratio, metabolic diseases

## Abstract

**Purpose:**

Our study aimed to assess the relationship between serum adipokines and insulin resistance (IR) in women with polycystic ovary syndrome (PCOS) and explore the predictive value of adipokines on IR in PCOS.

**Methods:**

This was a prospective cross-sectional study. 154 women with PCOS were included from July 2021 to September 2022 who underwent gonadal steroid hormone measurement, lipid profile, oral glucose tolerance test and homoeostasis model assessment (HOMA)-IR. Adiponectin (APN), leptin and secreted frizzled-related protein (Sfrp5) were measured by immunoturbidimetry and enzyme-linked immunosorbent assay. Women with PCOS were categorised based on the presence of IR.

**Results:**

Women with PCOS with IR (*n* = 99) had significantly lower APN level and APN-to-leptin ratio (A/L ratio) than those without IR (*n* = 55), whereas serum levels of leptin and Sfrp5 were similar between the two groups. In multivariable linear regression analysis, serum log (APN) and log (A/L ratio) were associated with log (HOMA-IR), and the association was statistically significant after adjusting for body mass index and free androgen index. The area under the receiver operating characteristic curve (95% CI) for APN and A/L ratio was 0.726 (0.644–0.807; *P* < 0.001) and 0.660 (0.569–0.751; *P* < 0.01), respectively, with cutoff values of 5.225 mg/L (Youden index ¼ 0.364) and 1.438 mg/L (Youden index ¼ 0.265).

**Conclusion:**

Our study demonstrated that serum APN was negatively related to IR. Serum APN may be useful as a clinical marker for IR in women with PCOS. Our findings warrant further investigations into the function of APN in the pathogenesis of IR in women with PCOS.

## Introduction

Polycystic ovary syndrome (PCOS) is characterised by oligoanovulation, polycystic ovarian morphology and elevated androgen levels. It is a common endocrinopathy, affecting 11–13% of women of reproductive age (1, 2). As a leading cause of anovulation in infertile women, PCOS remains a condition with multiple aetiologies that are yet to be completely understood, along with considerable metabolic consequences. Adipose tissue secretes a diverse array of signalling molecules that regulate key homoeostatic systems, including nutrient intake, energy expenditure, insulin secretion and insulin function. Among these secreted factors, cytokines and adipokines, such as tumour necrosis factor-alpha, C-reactive protein, interleukin-6, adiponectin (APN) and ghrelin play crucial roles in modulating immune and inflammatory responses (3, 4). Research has demonstrated that the expression profile of adipose tissue is altered in women with PCOS, leading to an imbalanced secretion of adipokines, which in turn negatively impacts the endocrine and reproductive systems. This dysregulated secretion, coupled with heightened inflammatory responses and insulin resistance (IR), may contribute to the pathogenesis of PCOS and its associated clinical manifestations (5, 6).

Metabolic abnormalities in women with PCOS include obesity, IR, dyslipidaemia and hypertension, which further increase susceptibility to diabetes and cardiovascular diseases, resulting in increased long-term morbidity and mortality rates (7). IR is one of the main pathophysiological mechanisms underlying the occurrence and development of PCOS and is the central link of metabolic abnormalities (8, 9); as such, early identification of IR in women with PCOS is important to prevent metabolic-related adverse health events (8, 10).

IR refers to a decrease in the efficiency of glucose uptake and utilisation in response to insulin and is prevalent in approximately 40–65% of women with PCOS (11, 12, 13). However, the mechanism linking PCOS to IR is not completely understood. Many studies have found that IR can be detected by various techniques, such as homoeostasis model assessment (HOMA)-IR, normal blood glucose insulin clamp technique, quantitative insulin sensitivity test index and other direct or indirect detection methods (14). The triglyceride/glucose index is also an effective tool for the early evaluation of IR (15). However, these detection technologies are time-consuming and expensive and require specialised equipment; moreover, they cannot be implemented on a large scale for early IR screening, especially in low-income areas. In this regard, a convenient and potential biochemical indicator should be identified for early diagnosis of IR.

Adipocytes are active endocrine secretory cells that produce cytokines, including APN, leptin, secreted frizzled-related protein (Sfrp5) and other adipokines, which play important roles in the development of PCOS (16, 17, 18). APN, leptin and Sfrp5 are adipokines that are essential to the regulation of metabolic homoeostasis, and they also affect insulin sensitivity (19). Some studies showed that women with PCOS have an elevated serum level of leptin and decreased levels of APN and Sfrp5 compared with healthy women of reproductive age (16, 20, 21). At the same time, some evidence suggested that the APN-to-leptin ratio (A/L ratio) is implicated as a stronger indicator of IR than individual adipokines, and it was even speculated as a measure of IR and a marker of PCOS (22, 23). The A/L ratio might help identify women at risk of developing metabolic abnormalities such as IR. The measurement of serum adipokines is relatively simple and time-saving, and some studies have revealed the relationships between adipokines and IR in women with PCOS. Determining one or several adipokines to predict IR specifically and sensitively is crucial for the early identification of IR.

Therefore, the aim of this study was to systematically assess the relationship between serum adipokines and IR and calculate the A/L ratio to explore the predictive value of adipokines on IR in women with PCOS.

## Materials and methods

### Study design and participants

This study screened 189 female patients aged between 18 and 40 years and diagnosed with PCOS from July 2021 to September 2022 in the Reproductive Medicine Centre of Shunde Hospital of Southern Medical University (The First People’s Hospital of Shunde, Guangdong, China). The patients were diagnosed with PCOS in accordance with the Rotterdam definition, which states that two of three criteria should be met (24): oligomenorrhoea or amenorrhoea, clinical hyperandrogenism (presence of hirsutism, androgenic alopecia and acne) or biochemical hyperandrogenism (based on the normal range of serum total testosterone (TT)), and characteristic imaging of polycystic ovaries (at least one ovary containing 12 or more peripheral follicles measuring 2–9 mm in diameter and/or ovarian volume of at least 10 mL) on transvaginal or abdominal ultrasound images. Participants were excluded based on the following criteria: pregnancy or lactation state, history of hyperandrogenic states (such as non-classical congenital adrenal hyperplasia, androgen-secreting tumours, Cushing’s syndrome or 21-hydroxylase deficiency), and use of any hormonal medication (including oral contraceptives, insulin-sensitising agents, antidiabetic medications, antiandrogens or glucocorticoids) for at least 3 months preceding the evaluation. All the participants had normal levels of thyroid-stimulating hormone (TSH) and prolactin.

The ethical committee of Shunde Hospital, Southern Medical University, approved this study (20210703). Patients agreed to participate and signed informed consent forms.

### Protocol

All subjects completed a uniform questionnaire to provide information regarding age, race, detailed menstrual cycle history, history of medication and other socio-demographic data, which were further reviewed at consultation.

Anthropometric parameters including height, weight, waist circumference (WC) and hip circumference (HC) were measured. The standing height and body weight of the participants were measured without shoes and heavy clothes by using a calibrated scale to the nearest 0.5 cm and 0.1 kg, respectively. The body mass index (BMI) was calculated by dividing the weight in kilograms by the square of the height in metres (25). WC was measured at the midpoint between the lowest rib and the iliac crest, and HC was determined over the widest portion of the gluteal and greater trochanteric region (26). The waist-to-height ratio (WHR) was calculated using the formula WC (cm)/HC (cm) (25). The polycystic ovarian morphology was assessed by transvaginal ultrasonography or abdominal ultrasonography (Aloka F-37 Ultrasound System with 8 MHz endovaginal transducer, Hitachi High-Tech Co., Japan).

Fasting blood samples were obtained in the morning after a 12-h overnight fast and used to measure hormonal and metabolic parameters. Patients were generally tested for basal follicle-stimulating hormone (FSH), basal luteinising hormone (LH), oestradiol (E_2_), progesterone (P), basal prolactin (PRL), TT, anti-Müllerian hormone (AMH), sex hormone-binding globulin (SHBG), total cholesterol (TC), APN, high-density lipoprotein cholesterol (HDL-C), low-density lipoprotein cholesterol (LDL-C), triglyceride (TG), TSH, free triiodothyronine (FT3) and free thyroxine (FT4). The remaining serum samples were stored at −80 °C for the measurement of adipokines, including APN, leptin and Sfrp5. The oral glucose tolerance test (75 g glucose) was performed, and glycaemia and insulinaemia were measured at 0 and 120 min, respectively. The patients were screened for Cushing’s syndrome and androgen-secreting neoplasms when clinically indicated during follow-up.

The free androgen index (FAI) was calculated as the ratio of TT levels (in nmol/L) to SHBG levels (in nmol/L) multiplied by 100(%) (27). Non-high-density lipoprotein cholesterol (non-HDL-C) was calculated as TC–HDL-C. HOMA-IR was determined using the following formula, where the levels of fasting insulin and glucose are expressed in μIU/mL and mmol/L, respectively: HOMA-IR = fasting insulin × fasting glucose/22.5. Subjects were considered to have IR if the HOMA-IR index was ≥2.6 × 10^−6^ mol U/L^2^ (28). The A/L ratio was calculated as the ratio of APN to leptin levels (in ng/mL) (29).

Patients diagnosed with PCOS were stratified into two groups based on the presence of IR.

### Laboratory measurements

This study conducted all biochemical measurements in the Department of Clinical Laboratory or in the central laboratory of the Shunde Hospital of Southern Medical University (The First People’s Hospital of Shunde). Automated chemiluminescent immunoassay kits (DxI 800 Access Immunoassay System, Beckman Coulter Inc., USA) were used to measure FSH, LH, E_2_, P, PRL, TT and SHBG. The intra- and inter-assay coefficients of variation of different parameters were as follows: <4.3 and 5.6% for FSH, <5.4 and 6.4% for LH, <7.7 and 4.7% for E_2_, <9.19 and 9.57% for P, <3.93 and 7.08% for TT, <1.61 and 6.92% for PRL and <4.8 and 5.5% for SHBG. AMH was measured using a chemiluminescent immunoassay diagnostic kit (Guangzhou Kangrun Biotech Co., Ltd, China). The plasma glucose concentration was measured by the hexokinase method (Beckman Glucose Analyzer II, Beckman Coulter Inc., USA). The plasma insulin concentration was determined by chemiluminescent immunoassay (insulin test, Mindray Biomedical Co., Ltd, China). The total coefficient of variation was <10%. Commercial enzymatic kits (Zybio Inc., China) were used to measure TC, TG, HDL and LDL. APN was measured by immunoturbidimetry using commercial kits (Zybio Inc., China). The total coefficient of variation was <10%. Automated chemiluminescent immunoassays (DxI 800 Access Immunoassay System, Beckman Coulter Inc., USA) were used to measure TSH, FT3 and FT4. The total coefficient of variation was <8%.

The serum levels of adipokines including leptin and Sfrp5 were measured using respective commercial enzyme immunoassay kits: leptin, ml028534V Human LEP ELISA Kit (Shanghai Enzyme-Linked Biotechnology Co., Ltd, China), with minimum detectable dose (MDD) = 0.5 ng/mL; Sfrp5, ml058391V Human Sfrp5 ELISA Kit (Shanghai Enzyme-Linked Biotechnology Co., Ltd, China), with MDD = 1.5 ng/mL. The total intra- and inter-assay coefficients of variation were <15 and <10%, respectively.

### Statistical methods

All statistical analyses were performed using SPSS version 26.0 software (https://www.ibm.com/account/reg/cn-zh/signup?formid=urx-19774) (SPSS Inc., USA). Data were presented as mean ± standard deviation or as median (interquartile range) for continuous variables. Differences in continuous variables between the two groups were analysed using the Student’s *t*-test for those with normal distribution and Mann–Whitney U test for those with skewed distribution. Median-scaled data for APN, leptin and Sfrp5 were log-transformed prior to multivariable linear regression analysis to determine the associations between HOMA-IR (log-transformed) and adipokines (log-transformed). For multivariable linear regression, no variables were adjusted in model 1, whereas BMI and FAI were adjusted in model 2. Receiver operating characteristic (ROC) analysis was conducted for adipokines and A/L ratio to evaluate their abilities to correctly discriminate IR and determine the cutoff point for the prediction of IR in women with PCOS. All *P* values were two-sided, and *P* < 0.05 was considered statistically significant.

## Results

After exclusion of 35 patients who did not meet the eligibility criteria of the study, 154 women with PCOS were included in this study. Among them, 99 (64.3%) were diagnosed with IR. None of the enrolled patients were alcoholics or smokers.

Statistically significant differences were found in BMI (23.83 (21.67–26.36) vs 21.09 (18.93–22.46), *P* < 0.001), SHBG (34.60 (21.40–67.10) vs 58.50 (35.20–94.00), *P* < 0.01) and FAI (6.20 (2.75–12.41) vs 4.38 (1.80–8.47), *P* < 0.01) between the IR group and the group without IR. Age, WC, WHR, FSH, LH, LH/FSH ratio, E_2_, P, TT and AMH were not significantly different between the IR group and the group without IR (Table 1).

**Table 1:** Differences in basal and endocrine characteristics of women with PCOS categorised based on the presence of IR.

	Insulin resistance		*P* value
	No (*n* = 55)	Yes (*n* = 99)	
Age (years)	26.82 ± 4.72	26.90 ± 4.58	0.917
BMI (kg/m^2^)	21.09 (18.93–22.46)	23.83 (21.67–26.36)	<0.001
WC (cm)	78.00 (70.00–82.00)	76.00 (70.00–82.00)	0.693
WHR	0.85 (0.77–0.94)	0.82 (0.74–0.89)	0.059
Hormonal profile			
FSH (IU/L)	6.09 ± 2.06	6.49 ± 1.58	0.184
LH (IU/L)	8.35 (4.86–12.67)	9.24 (6.73–13.64)	0.207
LH/FSH ratio	1.33 (0.76–2.15)	1.43 (0.93–2.40)	0.511
E_2_ (pg/ml)	35.88 (28.23–48.09)	38.29 (30.22–46.79)	0.824
P (pg/ml)	0.54 ± 0.28	0.53 ± 0.27	0.850
TT (nmol/L)	2.32 ± 0.90	2.43 ± 0.87	0.443
SHBG (nmol/L)	58.50 (35.20–94.00)	34.60 (21.40–67.10)	0.003
Free androgen index (%)	4.38 (1.80–8.47)	6.20 (2.75–12.41)	0.006
AMH (ng/ml)	6.60 (4.59–10.54)	7.59 (5.15–10.04)	0.532

Note: Values are expressed as mean ± standard deviation or median (interquartile range), unless stated otherwise.

Abbreviations: PCOS, polycystic ovary syndrome; IR, insulin resistance; BMI, body mass index; WHR, waist-to-height ratio; FSH, follicle-stimulating hormone; LH, luteinising hormone; E_2_, oestradiol; P, progesterone; TT, total testosterone; SHBG, sex hormone-binding globulin; AMH, anti-Müllerian hormone.

The metabolic characteristics of women with PCOS who were categorised based on the presence of IR are presented in Table 2. The group with IR had significantly higher levels of fasting glucose, fasting insulin, HOMA-IR and triglycerides than the group without IR (*P* < 0.05). Serum levels of LDL-C, HDL-C and non-HDL-C were similar between the two groups. The serum levels of adipocytokines including APN, leptin and Sfrp5 are also shown in Table 2. Women with PCOS and IR had significantly lower levels of APN and A/L ratio than those without IR (*P* < 0.05), whereas serum levels of leptin and Sfrp5 were similar between the two groups (Table 2).

**Table 2:** Differences in metabolic characteristics of women with PCOS categorised based on the presence of IR.

	Insulin resistance		*P* value
	No (*n* = 55)	Yes (*n* = 99)	
Plasma glucose and insulin levels			
Fasting glucose (mmol/L)	5.13 (4.94–5.38)	5.28 (5.05–5.65)	0.020
Fasting insulin (μIU/ml)	7.71 (6.01–9.77)	16.05 (13.67–22.60)	<0.001
HOMA-IR (10^−6^ mol U/L^2^)	1.80 (1.37–2.27)	3.87 (3.11–5.73)	<0.001
Lipid levels			
Triglycerides (mmol/L)	0.82 (0.70–0.98)	1.45 (1.17–2.03)	<0.001
LDL cholesterol (mmol/L)	2.47 ± 0.71	2.63 ± 0.65	0.171
HDL cholesterol (mmol/L)	1.32 ± 0.33	1.22 ± 0.34	0.081
Non-HDL cholesterol (mmol/L)	3.40 ± 1.29	3.50 ± 0.98	0.606
Adipocytokines			
Adiponectin (mg/L)	6.48 ± 2.08	4.94 ± 1.67	<0.001
Leptin (ng/ml)	4.30 ± 1.15	4.22 ± 1.27	0.716
Sfrp5 (ng/ml)	12.46 ± 4.09	12.80 ± 3.96	0.616
A/L ratio	1.62 ± 0.63	1.27 ± 0.54	<0.001

Note: Values are expressed as mean ± standard deviation or median (interquartile range), unless stated otherwise.

Abbreviations: PCOS, polycystic ovary syndrome; IR, insulin resistance; HOMA-IR, homoeostasis model assessment-insulin resistance; LDL, low-density lipoprotein; HDL, high-density lipoprotein; Sfrp5, secreted frizzled-related protein; A/L, adiponectin/leptin.

Multivariable linear regression analysis was performed to explore the associations between APN, leptin, Sfrp5 and A/L ratio with HOMA-IR. In model 1 without adjustment for any confounding factors, serum log (APN) was associated significantly with log (HOMA-IR), and the standardised coefficient (95% CI) was −0.43 (−0.93 to −0.46, *P* < 0.001). The log (A/L ratio) was associated significantly with log (HOMA-IR), and the standardised coefficient (95% CI) was −0.37 (−0.66 to −0.28, *P* < 0.001). When further adjustment for BMI and FAI was conducted in model 2, serum log (APN) and log (A/L ratio) were still significantly associated with log (HOMA-IR), and the standardised coefficients (95% CI) were −0.32 (−0.76 to −0.29, *P* < 0.001) and −0.29 (−0.55 to −0.18, *P* < 0.001), respectively (Table 3).

**Table 3:** Association of adipokines with insulin resistance in women with PCOS.

Variable	Linear regression of log (adipokines) on log (HOMA-IR)		
	Standardised coefficient	95% CI	*P* value
Model 1			
Adiponectin	−0.43	−0.93–−0.46	<0.001
Leptin	0.06	−0.20–0.45	0.453
Sfrp5	0.13	−0.05–0.53	0.105
A/L ratio	−0.37	−0.66–−0.28	<0.001
Model 2			
Adiponectin	−0.32	−0.76–−0.29	<0.001
Leptin	0.06	−0.18–0.43	0.419
Sfrp5	0.14	−0.01–0.52	0.055
A/L ratio	−0.29	−0.55–−0.18	<0.001

Note: Model 1 was not adjusted. Model 2 was adjusted for BMI and FAI.

Abbreviations: PCOS, polycystic ovary syndrome; HOMA-IR, homoeostasis model assessment-insulin resistance; CI, confidence interval; Sfrp5, secreted frizzled-related protein; A/L, adiponectin/leptin.

**Fig. 1:**
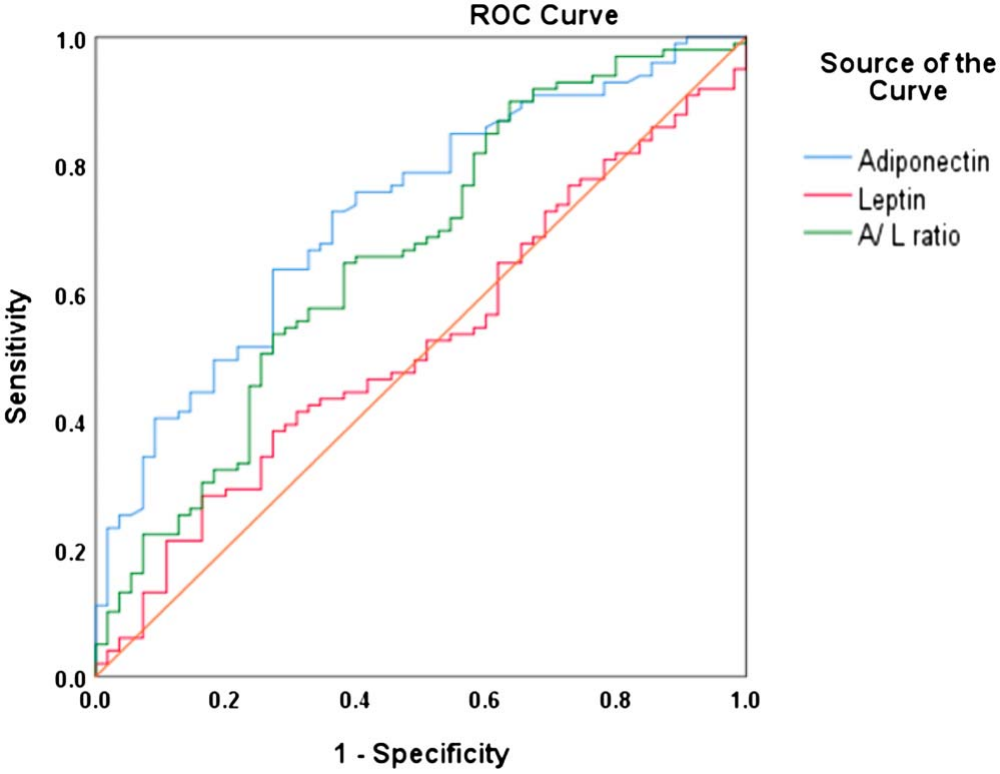
Receiver operating characteristic (ROC) curves and area under ROC curves for the detection of insulin resistance using adiponectin, leptin and A/L ratio.

APN, leptin and A/L ratio were evaluated as diagnostic markers for IR in women with PCOS. Sensitivity, specificity and AUC (area under curve) of APN, leptin and A/L ratio are shown in Table 4 and Fig. 1. The AUCs of APN and leptin were 0.726 (0.644–0.807; *P* < 0.001) and 0.523 (0.430–0.617; *P* = 0.631), respectively, with cutoff values of 5.225 mg/L (Youden index ¼ 0.364) and 3.147 ng/mL (Youden index ¼ 0.119). The ROC curve also showed a significant correlation between the A/L ratio and IR, with AUC of 0.660 (0.569–0.751; *P* < 0.01) and a cutoff value of the A/L ratio ≤ 1.438 (Youden index ¼ 0.265). APN showed the highest sensitivity for predicting IR in women with PCOS. We found no correlation between leptin and IR in women with PCOS.

**Table 4:** Evaluation of adiponectin, leptin and A/L ratio as a diagnostic test for IR in women with PCOS.

	Adiponectin	Leptin	A/L ratio
Area under the curve	0.726	0.523	0.660
Standard error	0.041	0.048	0.046
Sensitivity	63.6%	28.3%	64.6%
Specificity	72.7%	83.6%	61.8%
Cutoff	5.225	3.147	1.438
*P* value	<0.001	0.631	0.001
95% CI	0.644–0.807	0.430–0.617	0.569–0.751

## Discussion

Our study demonstrated that the serum APN level was negatively correlated with IR, thereby revealing that it was a protective factor of IR in women with PCOS. Furthermore, the serum APN level lower than 5.225 mg/L had a robust capability to predict IR.

Some clinical studies indicated that decreased plasma APN levels were associated with obesity, type 2 diabetes mellitus and cardiovascular diseases (30, 31, 32). These studies mainly focused on patients who have developed metabolic diseases. However, IR can be detected long before the development of metabolic diseases (33). Other studies also evaluated the relationship between APN and IR in PCOS, and a lower serum APN level was negatively related to IR in women with PCOS, suggesting the critical role of APN in IR development in PCOS (34, 35, 36). The results were consistent with our research findings, but they did not explore markers to predict IR in PCOS. In addition, the messenger RNA expression of leptin and APN decreases with increasing BMI. The APN and leptin expression were significantly low in women with PCOS. APN plays a critical role in insulin-sensitising. The decreased expression of APN may affect energy balance, insulin signalling and the regulation of reproductive hormones, all of which are core components of the pathophysiology of PCOS, highlighting APN’s importance in PCOS pathogenesis (37, 38, 39). Sepilian, Yildiz and coworkers also found a significant negative correlation between serum APN levels and IR in women with PCOS, but they employed a small sample size and conducted only a simple correlation analysis of serum APN level and IR in their studies (40, 41). In contrast, a linear regression model was used in the present study to reflect the correlation strength of APN with IR. The results indicate that the association between the serum APN level and IR still existed after the confounding factors were adjusted, thereby eliminating the role of BMI and FAI in altering these adipokines. Consequently, our study confirmed that APN is a protective factor against IR in women with PCOS.

Although the mechanism of APN leading to IR remains unknown, many studies found that increasing the circulating levels of APN through exogenous administration enhances insulin sensitivity and inhibits gluconeogenesis (42, 43). Moreover, the systemic deletion of APN tends to induce diabetes (44, 45). As a result, it exhibits a more negative metabolic phenotype than the congenital knockout of APN in mature adipose tissues (46). APN can also enhance exosome biogenesis and secretion, which contributes to a reduction in cellular ceramide levels. High levels of ceramides are recognised as contributors to IR (47).

Animal studies also showed that 2 weeks of globular APN treatment reduces fasting plasma glucose, triglyceride and insulin levels, and whole-body IR is reversed (48). Another study showed that exogenous APN supplementation in early pregnancy normalises the PCOS-like endocrine phenotype of adult female offspring mice with PCOS; the authors found that this treatment significantly corrects metabolic disorders, including obesity, IR, impaired glucose tolerance and hyperlipidaemia, thereby suggesting that APN intervention can be a novel therapy for IR and metabolic syndrome in women with PCOS (49). Furthermore, the overexpression of APN is a protection against the development of metabolic disturbances in PCOS-like mouse models (50). All these experimental findings provide evidence that the occurrence and development of IR are related to APN; moreover, APN may play a significant role in determining insulin sensitivity (47, 51). Our study demonstrated that APN could serve as an important biochemical indicator for the diagnosis of IR in women with PCOS. Therefore, APN may be used as a predictor for the treatment of IR and metabolic syndrome in women with PCOS in the future.

The low A/L ratio, which is independently associated with PCOS, was proposed to be a novel marker for obesity and IR (22, 23, 29). Our results were similar to the findings of these previous studies showing that the level of A/L ratio in women with PCOS with IR is significantly lower than that in women with PCOS without IR. However, our ROC curve showed higher sensitivity and specificity of APN for the diagnosis of IR in women with PCOS with a larger area under the curve relative to A/L ratio. Thus, we concluded reasonably that the APN may be an effective predictor of IR in women with PCOS.

Our study has a few limitations. First, we based our diagnosis of IR on HOMA-IR rather than on the hyperinsulinaemic-euglycaemic clamp, which is the gold standard for evaluating insulin sensitivity. However, this method is invasive, and some studies have shown a good correlation between HOMA-IR and the hyperinsulinaemic-euglycemic clamp (15, 52). Second, the testosterone levels were measured in our study using a chemiluminescent immunoassay kit, which has limited accuracy and poor sensitivity for diagnosing hyperandrogenism in PCOS compared to liquid chromatography-mass spectrometry/mass spectrometry. Third, the cross-sectional design of the study has limitations for the interpretation of the causality of associations. Finally, the conclusion needs to be verified in future studies with a large sample size.

## Conclusion

Our study demonstrated that the serum APN level was negatively related to IR and the serum APN level lower than 5.225 mg/L had a robust capability to be a marker of IR in women with PCOS. Thus, APN may be valuable for early identification of IR in PCOS, and it is of great importance to prevent metabolic-related adverse health events in the future.

## Declaration of interest

The authors declare that there is no conflict of interest that could be perceived as prejudicing the impartiality of the work reported.

## Funding

This work was supported by the Guangdong Basic and Applied Basic Research Foundation (grant no. 2022A1515140094), China.

## Author contribution statement

J Yang and X Chen helped in project development, data collection and manuscript writing. M Lin and X Li contributed to literature searching, data analysis and article revision. C Li and L Deng helped in data collection and manuscript editing. X Tian and H Wu contributed to data collection. All authors approved the final version of the manuscript.

## Data availability

All data generated or analysed in the present study are included in this published article.

## Ethics approval consent to participate

The approval for this study was obtained from the Ethical Committee of Shunde Hospital, Southern Medical University (20210703). The patients recruited in this study have all agreed to participate and signed informed consent forms.

## Consent for publication

Written informed consent for publication was obtained from the patients.
